# Study on the relationship between Methylation of Neuregulin (NRG) Gene and Cervical Carcinoma

**DOI:** 10.12669/pjms.40.6.7859

**Published:** 2024-07

**Authors:** Xizhao Yan, Fenglan An, Zhenzhen Ding, Dong Ma, Xiaohui Yang

**Affiliations:** 1 Xizhao Yan, Department of Gynaecology, Cangzhou People’s Hospital, Cangzhou 061000, Hebei, China; 2 Fenglan An, Radiation and Chemotherapy Division, Cangzhou Hospital of Integrated TCM-WM, Hebei, Cangzhou 061000, Hebei, China; 3 Zhenzhen Ding, Department of Gynaecology, Cangzhou People’s Hospital, Cangzhou 061000, Hebei, China; 4 Dong Ma, School of Public Health, North China University of Science and Technology, Tangshan 063210, Hebei, China; 5 Xiaohui Yang, Science and Technology Experimental Center, Cangzhou Medical College, Hebei, China, Cangzhou 061001, Hebei, China

**Keywords:** Neuregulin, Cervical carcinoma, DNA methylation, Real-time quantitative PCR, Immunohistochemistry

## Abstract

**Objective:**

To investigate the relationship between the DNA methylation state of NRG1 promoter and its expression changes, and to analyze the clinical significance of its regulatory mechanism of DNA methylation in cervical carcinoma.

**Methods:**

This was a retrospective study. One-hundred and twenty patients from the Department of Gynecology of Cangzhou People’s Hospital from September 2017 to September 2019 were selected, including 40 cases of cervical SCC, 40 cases of high grade squamous intraepithelial lesions(HSIL) and 40 cases of control cervical tissues. RT-qPCR, immunohistochemistry and DNA methylation-specific PCR(MSP) were used to detect the mRNA and protein expression of NRG1 and DNA methylation status in different tissue types.

**Results:**

Immunohistochemical results showed that the positive protein expression rate of NRG1 gene in the SCC group was lower than that in both HSIL and Control groups. RT-qPCR results showed that the mRNA gene of NRG1 gradually decreased in expression with the increase of cervical tissue lesions, with a statistically significant difference. Similarly, it also found that the mRNA expression level of NRG1 in the SCC group was independent of patients’ age (p>0.05), but significantly correlated with tumor pathological staging, surgical pathology staging and lymphatic metastasis (p<0.05). Furthermore, methylation-specific PCR results revealed a significantly higher DNA methylation rate of NRG1 gene in the SCC group than in both HSIL and Control groups, with a statistically significant difference. Moreover, the methylation degree of NRG1 gene in SCC tissues was negatively correlated with its mRNA expression (p<0.05).

**Conclusions:**

Abnormal DNA hypermethylation of NRG1 gene inhibits the expression of mRNA and protein in the progression of cervical tissue from normal to cancerous state, which is involved in the occurrence and development of cervical carcinoma.

## INTRODUCTION

Cervical carcinoma is one of the most common malignancies threatening women’s health worldwide, with an etiology attributable to persistent infection with high-risk human papillomavirus(HPV).^1^ China, as the largest developing country in the world, accounts for about 1/4 of all new cases of cervical carcinoma in the world each year.^2^,^3^ Despite the efforts made in the screening and prevention of HPV in recent years, HPV in China has been hovering at a high level of incidence and mortality^4^ and tends to make inroads on young people. Meanwhile, there are still debates and gaps regarding the pathogenesis of cervical carcinoma. To this end, the potential molecular mechanisms underlying the development of cervical carcinoma should be clarified and the potential molecular targets for its diagnosis and treatment should be identified, which is of great practical importance to improve the quality of survival and prognosis of patients with cervical carcinoma.

Recent years have witnessed a spurt of progress in genomics and modern molecular biology, and it is gradually recognized that epigenetic alterations, especially DNA methylation, are important indicators for detecting the occurrence and progression of cervical carcinoma.^5^-^7^ However, there are no studies on the role of NRG in cervical carcinoma. In this study, the protein and nucleic acid expression of NRG in different pathological stages of cervical carcinoma was analyzed, and thus the relationship between the DNA methylation state of NRG gene promoter region and its expression in different types of cervical carcinoma tissues was clarified.

## METHODS

This was a retrospective study. One-hundred and twenty patients from the Department of Gynecology of Cangzhou People’s Hospital from September 2017 to September 2019 were selected, including 40 cases of cervical SCC, 40 patients with pathologically confirmed tissues of HSIL who underwent outpatient biopsy or cervical cornization surgery were selected as the HSIL group, and 40 control cervical tissues (hysterectomy for benign lesions, excluding malignant cervical lesions by postoperative pathology) were selected as the Control group. All specimens collected were divided into two parts after being read and determined by pathologists. One was kept in the refrigerator at -80°C, and the other was fixed by neutral formalin and embedded in paraffin (for immunohistochemical experiments). Water and one placental tissue were also taken as negative and positive controls for methylation. Moreover, all patients were assured that they had not received radiation, chemical and hormonal treatment prior to surgery. All specimens were collected with the informed consent of the patients, and their histological types and grading were confirmed by 2 pathologists of associate chief physician or above.

### Ethical Approval:

The study was approved by the Institutional Ethics Committee of Cangzhou People’s Hospital (No.: CY-SYDW-2021-01; date: May 18, 2021), and written informed consent was obtained from all participants.

### Inclusion criteria:

- Patients with complete and coherent clinical and pathological data;
- Patients with pathological findings showing a confirmed diagnosis of SCC or HSIL;
- Patients who had not received radiotherapy, chemotherapy or hormone therapy prior to surgery.

### Exclusion criteria:

- Patients who had not received radiation, chemotherapy or hormone therapy within three months prior to surgery;
- Patients who were pregnant at the time of consultation or surgery;
- Patients with a combination of gynecologic malignancies such as ovarian cancer or a combination of other malignant tumors at other sites, such as gastric cancer and colorectal cancer.^7^

Total RNA Kit I R6834 and Tissue DNA Kit D3396 were purchased from OMEGA, while DNA Methylation Bisulfite Kit was purchased from Tiangen Biotech (Beijing) Co., Ltd. Besides, PCR primers (including Real-time qPCR primers and MSP primers) were synthesized by Sangon Biotech (Shanghai) Co., Ltd.

Immunohistochemical detection of the expression level of NRG in different types of cervical tissues: Firstly, the tissue sections were embedded in paraffin wax and sliced into thin sections continuously, and were subjected to the steps of xylene III, II, and I soaking, dehydration by anhydrous ethanol of different types and concentrations, antigen retrieval, natural cooling at room temperature, etc. Subsequently, color development and re-staining were performed according to the experimental steps of the staining kit. Finally, the tissue sections were sealed using neutral gum and observed directly under the microscope. In the same procedure, PBS was selected as the blank control instead of the primary antibody, whereas control cervical tissue slices were used as the positive control. The presence of brown staining in the cytoplasm was considered positive. The positive rate in the whole experimental group was calculated.

### PCR detection of mRNA expression level of target gene:

Total RNA was extracted as per the operating manual of Total mRNA Extract kit (Omega). RNA solution 2 μL (2.3.1) obtained was taken for quantification to confirm that the RNA was intact and free of degradation. 1 μg of total RNA was taken and reverse-transcribed into cDNA according to the operating manual of M-MLV First Strand Kit. The reverse-transcribed products were diluted according to the instructions in a ratio of 1:25, and the reaction system was prepared proportionally for quantitative PCR reaction and reverse transcription PCR reaction. And 1.5% agarose gel was used for constant pressure electrophoresis. The relative quantification of RNA was performed by the comparative Ct method. Specifically, GAPDH was selected as the internal reference gene, the ∆Ct value (Ct destination - Ct internal reference) was calculated for each group, and the 2-∆Ct method was used to calculate the expression amount of the statistical data converted into the target RNA. For reliable experimental results, ultra-pure water was chosen as a blank control for each electrophoresis performed. Moreover, the electrophoretic results were observed under UV light and analyzed by gel electrophoresis image system (Table-I).

**Table-I:** Analysis of RT-PCR primers.

Gene	Sense	Anti-sense
GAPDH	CGTCCCGTAGACAAAATGGT	GAGGTCAATGAAGGGGTCG
NRG-1	CAAAGAAGGCAGAGGCAAAG	AACTGGTTTCACACCGAAGG

### Methylation-specific PCR(MSP):

The promoter sequence of NRG1 gene was analyzed using UCSC Genome Browser and its CpG islands were analyzed using Primer Premier 5.0 software. Two sets of PCR primers (methylation and non-methylation) were designed according to the results of promoter analysis and CpG islands, and then synthesized by Sangon Biotech (Shanghai) Co., Ltd. Referring to the laboratory requirements, tissue DNA was extracted according to certain steps, and bisulfite conversion was performed as per the operating manual of the DNA Bisulfite Conversion Kit from iangen Biotech (Beijing) Co., Ltd. Then the products amplified by PCR were mixed with the sample buffer and subjected to 120 V constant pressure electrophoresis on 1.5% agarose gel. For reliable experimental results, ultra-pure water was chosen as a blank control for each electrophoresis performed. The electrophoretic results were observed under UV light and analyzed by gel electrophoresis image system. Moreover, 70 μL of the products that showed methylation and unmethylation in the above MSP results were selected for sequence evaluation (Table-II).

**Table-II:** Methylated and unmethylated sequences of NRG1.

NRG1 primer	Primer sequence
NRG1-M	F: GGGGIAATTGAAAAAGAG
	R: ACCCACCTAAACTCTAACTACC
NRG1-U	F:GAGGGATAAATTTTTTTTAAAT
	R: CTATCCCTTACCCTAAACTCTAAAC

### Statistical analysis:

The data of clinical population was entered into Excel 2007 and a database of the original data of the samples was established. SPSS 21.0 software was used to statistically analyze the experimental data for comparison of differences between groups, with p<0.05 indicating a statistically significant difference. The mRNA expression results of NRG1 gene were expressed as *χ̅*±*S*, and the comparison of differences between groups for quantitative data was performed by independent samples t test, one-way ANOVA or Fisher’s exact probability method. In addition, the protein positive rate and methylation rate were expressed as percentage, and χ^2^ test was used to analyze the differences between groups for qualitative data.

## RESULTS

The general data of the patients was queried through electronic medical records system and recorded in Excel. Constituent factors of patients in the Control group, HSIL group and SCC group were compared(p>0.05), with no statistically significant differences. In this case, it was considered that none of the above factors in the target population of the three groups with different pathological types would have an impact on this experiment, indicating the comparability of the data of the experimental results, as shown in Table-III.

**Table-III:** General data questionnaire.

Basic characteristics	Control (N=40)	HSIL (N=40)	SCC (N=40)	P
Age (years)	50.03±8.02	48.37±4.80	47.50±3.69	0.261a
Age of marriage registration (years)	25.26±3.50	24.75±2.97	25.60±3.59	0.805a
Age of first menstruation (years)				
<12	13	14	16	0.834c
≥12	27	26	24	
Menstrual cycle (days)				
<26	7	12	11	0.841c
26-30	25	12	23
≥30	8	16	6
Menstrual days (days)				
<5	12	14	9	0.837c
5-7	19	14	25
≥7	9	12	6
Number of pregnancies (times)				
<3	24	26	18	0.281c
≥3	16	14	22	
Number of abortions (times)				
<2	26	28	31	0.282c
≥2	14	12	9	
Smoking (number of cases)				
Yes	6	13	7	0.835b
No	34	27	33	
Alcohol consumption (number of cases)				
Yes	13	26	15	0.724b
No	27	14	25	

***Note:*** A represents analysis of variance, b represents c^2^ test, and c represents Fisher’s exact probability method.

The positive protein expression rate of NRG1 gene in the SCC group was lower than that in both HSIL and Control groups, and the positive expression rate gradually decreased with the aggravation of cervical lesions. It was found by statistical analysis of clinical data that NRG1 protein expression in the SCC group was independent of patients’ age(p>0.05), but significantly correlated with clinicopathological staging, surgical pathology staging, and lymphatic metastasis (p<0.05), as shown in Table-IV.

**Table-IV:** Comparison of mRNA expression rates of NRG1 in different cervical carcinoma tissues.

Group	N	NRG-1 expression		

		Positive (N)	Negative (N)	Positive rate (%)
Control	40	26	14	65.0
HSIL	40	20	20	50.0
SCC	40	4	36	10.0

c^2^=25.748, p=0.01, with a statistically significant difference.

**Fig.1:**
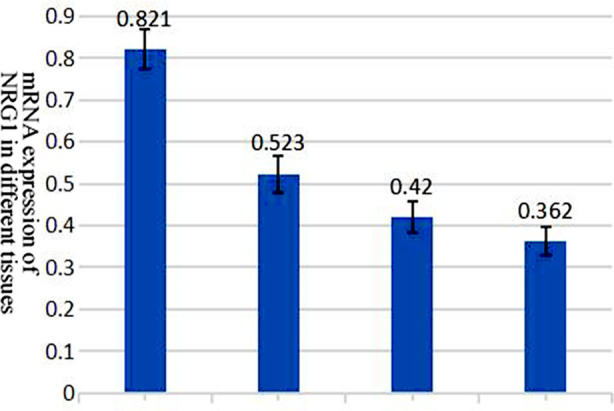
mRNA expression of NRG1 in cervical tissues in different groups.

mRNA expression of NRG1 in cervical tissues in different groups. The mRNA gene of NRG1 gradually decreased in expression with the increase of cervical tissue lesions (p<0.05), with a statistically significant difference. The mRNA gene of NRG1 in the HSIL group was statistically significantly different from that in the Control group after comparison(p<0.01), while that in the SCC group was statistically significantly different from that in the Control group after comparison (p<0.01). Similarly, statistical analysis of clinical data also found that the mRNA expression level of NRG1 in the SCC group was independent of patients’ age (p>0.05), but significantly correlated with lymphatic metastasis, tumor clinical staging, and surgical pathology staging(p<0.05), as shown in Fig.1 and Table-VI.

**Table-V:** Relationship between protein expression in endometrial carcinoma tissues and clinicopathological characteristics of patients.

	N	Positive expression of NRG protein		c^2^	P

		N	Positive rate (%)		
***Age***					
≥50	11	7	63.6	0.012	0.911
<50	29	19	65.5		
***Surgical pathology staging***					
I	21	10	47.6	6.614	0.037
II	14	11	78.5		
III	5	5	100.0		
***Histopathological staging***					
G1	15	6	40.0	8.397	0.015
G2	12	8	66.6		
G3	13	12	92.3		
***Metastasis or not***					
Yes	13	12	92.3	6.313	0.012
No	27	14	51.8		

**Table-VI:** Relationship between mRNA expression of NRG1 in cervical carcinoma tissues and clinicopathological features of patients.

	N	Expression of NRG protein	P
***Age***			
≥50	11	0.344±0.155	0.375b
<50	29	0.384±0.114	
***Surgical pathology staging***			
I	21	0.484±0.045	0.014a
II	14	0.306±0.047	
III	5	0.189±0.039
***Histopathological staging***			
G1	15	0.457±0.085	0.032a
G2	12	0.254±0.052	
G3	13	0.172±0.033	
***Metastasis or not***			
Yes	13	0.442±0.039	0.016b
No	27	1.053±0.042	

***Note:*** A represents one-way ANOVA, and b represents t test; p<0.05 indicates a statistically significant difference.

Methylation-specific PCR results revealed a significantly higher DNA methylation rate of NRG1 gene in the SCC group than in both HSIL and Control groups (p=0.01), with statistically significant differences between the groups. Moreover, the methylation rate increased sequentially with the aggravation of the lesion site, suggesting a possible correlation between the abnormal expression of NRG1 in cervical carcinoma tissues and the methylation status of this gene, as shown in Table-VII.

**Table-VII:** Comparison of methylation rates of NRG1 gene promoter in different cervical tissues.

Group	N	NRG-1 methylation		

		Positive (N)	Negative (N)	Positive rate (%)
SCC	40	29	11	72.5
HSIL	40	9	21	22.5
Control	40	1	39	4.3

Spearman’s correlation test was performed on the methylation status of NRG1 gene and mRNA expression, with a correlation coefficient (rs) of -0.740 (p<0.05). In other words, during the development of cervical tissues from normal state to precancerous lesions until the final cervical carcinoma, mRNA expression gradually decreased and the methylation rate increased successively, suggesting a correlation between the decreased expression of NRG1 in cervical carcinoma tissues and its increased DNA methylation rate, as shown in Table-VIII.

**Table-VIII:** Correlation analysis between mRNA expression and promoter methylation of NRG1 in cervical carcinoma tissues.

Group	Number of cases (N)	Methylation positive rate (%)	Relative expression of mRNA (2-ΔΔCt)	rs	p
Control	40	2.5	1.28±0.14	-0.740	0.01
HSIL	40	21.4	0.67±0.10		
SCC	40	72.5	0.30±0.13		

## DISCUSSION

In this study, cervical pathological tissue specimens were collected, and immunohistochemistry (ICH) and real-time fluorescence quantitative polymerase chain reaction (RT-qPCR) were employed to detect the protein level and mRNA expression of NRG in cervical carcinoma and its precancerous tissues. The MSP method was used to detect the methylation status of NRG1 in cervical carcinoma group, precancerous lesion group and control tissue group, and to analyze the relationship between the methylation status of NRG1 and its expression. The results revealed a significantly higher DNA methylation rate of NRG1 gene in the SCC group than in both HSIL and Control groups, with a statistically significant difference by comparing the methylation rates between multiple groups of specimens. This indicated that the expression of NRG1 gene in cervical tissue had an effect on different types of cervical lesions, and the methylation rate was gradually increased with the aggravation of cervical lesions.

Meanwhile, the higher methylation of NRG1 gene in cervical carcinoma than in control and precancerous lesion groups also indicated that NRG1 is a negative regulator of cervical carcinoma cell growth, and the reduction or absence of its expression is a favorable condition for the development and deterioration of cervical carcinoma. In this study, the positive protein expression of NRG1 gene was firstly detected by immunohistochemical assay, and the results showed a lower protein positive expression rate of NRG1 gene in cervical carcinoma tissues than in both precancerous lesion and control groups. Subsequently, the mRNA expression of the NRG1 gene was examined at the molecular nucleic acid level by RT-qPCR experiments, and the results showed that the mRNA expression of NRG1 decreased gradually with the aggravation of lesions at the cervical site. This raises a question: Is there any correlation between the DNA methylation status of NRG1 gene and its mRNA expression? Theoretically, excessive DNA methylation of the gene would inhibit its transcription and thus lead to expression silencing. In this study, correlation analysis of NRG1 gene methylation and its mRNA expression showed a Spearman correlation coefficient (rs) of -0.740 (p<0.05), indicating that the mRNA expression of NRG1 gene decreased in patients during the development process from the normal state of cervical tissue to precancerous lesions and eventually carcinoma. Thus, it can be concluded that the hypermethylation of NRG1 gene may have a close bearing on the occurrence and development of cervical carcinoma.

Despite the correlation between cervical carcinoma and high-risk human papillomavirus (HR-HPV) infection confirmed in numerous studies, persistent HR-HPV infection is not sufficient to cause immortalization and transformation of cervical epithelial cells, where epigenetic alterations play a non-negligible role in the process of cervical carcinoma.^8^ NRGI has been shown in several foreign studies to be highly expressed in various tumors such as lung cancer and renal clear cell carcinoma.^9^ It plays a crucial role in several aspects of malignant tumor development and is involved in tumor proliferation growth, differentiation, metastasis, microangiogenesis and microenvironmental changes.^10^-^14^ However, no studies have been reported on whether changes in NRG1 expression are associated with cervical carcinoma.

On the contrary, recent studies in China have also identified NRG as an important class of intercellular signaling proteins that are involved in a variety of intercellular signaling. Related studies have revealed multiple pathways in which NRGs play important roles in multiple aspects of malignant tumor development. Neuregulins (NRGs), belonging to the EGF family, are signaling proteins that mediate interactions between cells. They are mainly expressed in brain, heart, breast and other tissues, and participate in the development and maturation of tissue cells. NRGs includes four members (NRG1-4), among which only NRGI has been studied in detail.^15^ NRG1 is an epidermal growth factor (EGF)-like signaling protein expressed in a variety of tissues that acts as a ligand to bind and activate members of the receptor tyrosine kinase family (ErbBs), thus mediating multiple intercellular signaling.^16^ Li Wei suggested that the mRNA expression of NRG1 gene was diminished in endometrial cancer tissues, and the expression decreased with the increase of pathological grading and the progress of surgical pathology staging. Accordingly, he speculated that NRG1 is likewise a negative regulatory factor for the growth of cervical carcinoma cells, and the reduction or absence of its expression is a favorable condition for the development and deterioration of cervical carcinoma.^17^

With the development of modern molecular biology and genomics, targeted therapy has become one of the research hotspots in the treatment of cervical carcinoma. In this study, methods such as MSP, immunohistochemistry and real-time fluorescence PCR were employed to analyze the methylation status, protein and nucleic acid expression of NRG in different pathological stages of cervical carcinoma, and a correlation analysis was conducted between NRG1 expression in cervical carcinoma and the clinical characteristics of patients with cervical carcinoma, patients endocervical neoplasia and normal population. The role of NRG in cervical carcinoma has been well interpreted from clinical samples to cytological studies.

This present study not only confirmed that NRG1 could be expressed in cervical carcinoma tissues, HSIL tissues and normal cervical tissues, but also showed its relatively low expression in cancerous tissues along with the aggravation of cervical site lesions. This, confirmed by RT-qPCR, also applies to the expression of NRG1 at the molecular nucleic acid level. It was also found by immunohistochemical studies combined with clinical statistics that the mRNA expression of NRG1 gene in cervical carcinoma tissues was independent of the patients’ age, but significantly correlated with lymphatic metastasis, tumor clinical staging, and surgical pathology staging. All of these suggest a role for NRG1 in the development of cervical carcinoma. However, no further investigation was conducted in this study regarding the mechanism by which NRG1 exerts the above content effects and the prognosis of patients with cervical carcinoma, etc.

DNA methylation, as a part and parcel of epigenetics, contributes much to the maintenance of normal cell function, genetic imprinting, embryonic development and human tumorigenesis. It refers to the covalent binding of methyl groups to the CpG cytosine ring, a process catalyzed by DNA methyltransferase. Numerous studies have shown the unique methylation pattern of tissue cell genomes in normal humans, which will undergo abnormal changes once tumor development occurs, such as the overall hypomethylation state of tumor cells and hypermethylation in specific regions.^18^ Hypermethylation of CpG islands has been shown to be highly correlated with transcriptional silencing of related genes, especially CpG islands in the promoter region. Hypomethylation of genes leads to gene instability, transposon reactivation, and activation of proto-oncogenes. In contrast, hypermethylation of specific regions of oncogenes during tumor development leads to silencing of related genes. Therefore, it is evident that abnormal DNA methylation plays a non-negligible role in the epigenetic mechanism of cervical carcinoma.^18^

Based on the above results, it can be inferred that NRG1 gene methylation occurs in cervical carcinoma tissues, which is likely to be a manifestation of cervical tumor specificity. This conclusion is further confirmed by the negative correlation (p<0.05) between the degree of DNA methylation of NRG1 gene and the expression of mRNA in cervical carcinoma shown in this study.

### Limitations:

However, this study has its limitations, including a small sample size and a short follow-up period. Consequently, the survival time and long-term therapeutic effects of this treatment regimen have not been included in the study. In further clinical work, we intend to increase the sample size, extend the follow-up time, and incorporate other drug combination regimens into the research to comprehensively and objectively evaluate the long-term effects of this treatment regimen on patients.

## CONCLUSION

DNA methylation of NRG1 gene may be involved in the occurrence and development of cervical carcinoma. If the DNA methylation status of NRG1 gene is identified as an epigenetic marker from an epigenetic point of view, molecular therapies for patients with cervical carcinoma can be used as a reference for the targeted therapy of cervical carcinoma.

### Authors’ Contributions:

**XY and FA** carried out the studies, participated in collecting data, drafted the manuscript, and are responsible and accountable for the accuracy and integrity of the work.

**ZD and** **DM** performed the statistical analysis and participated in its design.

**XY** participated in acquisition, analysis, or interpretation of data and draft the manuscript.

All authors read and approved the final manuscript.
